# Chloroplast genome characteristics and phylogeny of the *sinodielsia* clade (apiaceae: apioideae)

**DOI:** 10.1186/s12870-023-04271-2

**Published:** 2023-05-29

**Authors:** Long Weng, Yunhui Jiang, Yong Wang, Xuemei Zhang, Ping Zhou, Mei Wu, Hongzhe Li, Hang Sun, Shaotian Chen

**Affiliations:** 1 College of Traditional Chinese Medicine, Yunnan University of Chinese Medicine, Kunming, 650500 China; 2 Yunnan Institute of Forest Inventory and Planning, Kunming, 650051 China; 3 Yunnan Key Laboratory of Dai and Yi Medicines, Yunnan University of Chinese Medicine, Kunming, 650500 China; 4 College of Basic Medicine, Yunnan University of Chinese Medicine, Kunming, 650500 China; 5 Key Laboratory for Plant Diversity and Biogeography of East Asia, Kunming Institute of Botany, Chinese Academy of Sciences, Kunming, 650201 China

**Keywords:** *Sinodielsia* clade, Systematics, Cp genome, Highly variable region, Apioideae

## Abstract

**Background:**

The *Sinodielsia* clade of the subfamily Apioideae (Apiacieae) was established in 2008, and it is composed of 37 species from 17 genera. Its circumscription is still poorly delimited and unstable, and interspecific relationships in the clade lack comprehensive analysis. Chloroplast (cp.) genomes provide valuable and informative data sources for evolutionary biology and have been widely used in studies on plant phylogeny. To infer the phylogenetic history of the *Sinodielsia* clade, we assembled complete cp. genomes of 39 species and then performed phylogenetic analysis based on these cp. genome sequence data combined with 66 published cp. genomes from 16 genera relative to the Sinodielsia clade.

**Results:**

These 39 newly assembled genomes had a typical quadripartite structure with two inverted repeat regions (IRs: 17,599–31,486 bp) separated by a large single-copy region (LSC: 82,048–94,046 bp) and a small single-copy region (SSC: 16,343–17,917 bp). The phylogenetic analysis showed that 19 species were clustered into the *Sinodielsia* clade, and they were divided into two subclades. Six mutation hotspot regions were detected from the whole cp. genomes among the *Sinodielsia* clade, namely, *rbc*L–*acc*D, *ycf*4–*cem*A, *pet*A–*psb*J, *ycf*1–*ndh*F, *ndh*F–*rpl*32 and *ycf*1, and it was found that *ndh*F–*rpl*32 and *ycf*1 were highly variable in the 105 sampled cp. genomes.

**Conclusion:**

The *Sinodielsia* clade was subdivided into two subclades relevant to geographical distributions, except for cultivated and introduced species. Six mutation hotspot regions, especially *ndh*F–*rpl*32 and *ycf*1, could be used as potential DNA markers in the identification and phylogenetic analyses of the *Sinodielsia* clade and Apioideae. Our study provided new insights into the phylogeny of the *Sinodielsia* clade and valuable information on cp. genome evolution in Apioideae.

**Supplementary Information:**

The online version contains supplementary material available at 10.1186/s12870-023-04271-2.

## Background

Apioideae is the most complicated subfamily of the Apiaceae family in taxonomy, and this subfamily comprises approximately 380 genera and 3,200 species (Angiosperm Phylogeny Website, Stevens, updated 2021, https://www.mobot.org/MOBOT/research/APweb/). Its members are widely distributed all over the world [1]. Its earliest lineage appeared in Southern Africa, but representatives appeared more frequently in the north temperate zone of Eurasia [2, 3]. Apioideae is definitely monophyletic and subdivided into 16 tribes and 14 clades, but many tribes and clades of the subfamily are not monophyletic [4–6]. Furthermore, circumscriptions of some genera are poorly delimited, and species of these genera always cluster to more than one tribe or clade based on molecular phylogenetic studies [6]. Therefore, Apioideae is a puzzle in terms of classification and phylogeny.

The *Sinodielsia* clade was established as a novel clade in Apioideae based on nuclear ribosomal (nr) DNA internal transcribed spacer (ITS) sequence data in 2008 [7] and initially included 14 species from 10 genera of Apioideae (Table 1). Since then, the classification of the *Sinodielsia* clade has attracted considerable interest, and phylogenetic studies have spurred taxonomic realignments of relative genera so that an increasing number of species from other genera were transferred to this clade in view of nrDNA ITS and chloroplast (cp.) DNA data [8–10]. Currently, the *Sinodielsia* Clade comprises 37 species from 17 genera in total (Table 1), and some of them are important medicinal herbs with great economic value: *Angelica sinensis* (Oliv.) Diels, *Ligusticum sinense* Oliv., *Cnidium dahuricum* (Jacq.) Turcz. ex Fisch. & C.A. Mey., *Conioselinum vaginatum* (Spreng.) Thell., and so on [5, 7–10].

Studies on Apioideae systematics have contributed to the circumscription of the *Sinodielsia* clade in principle, except that several of its members seem to be controversial and indeterminate in systematic positions based on different datasets: *Peucedanum delavayi* Franch., *Ligusticum pteridophyllum* Franch. and *Seselopsis tianschanica* Schischkin [5, 8–13]. Therefore, it is necessary and useful to conduct a comprehensive analysis of the phylogeny of the clade and its relatives to confirm the interspecific relationships of the clade and obtain a better understanding of the evolution of the clade and subfamily.

Chloroplast genomes are characterized by a highly conserved structure, fewer gene arrangements, and relatively coincident gene contents among plant species [14]. Furthermore, because developments in next-generation sequencing (NGS) and improvements in algorithms have decreased the cost of data acquisition and complexity of the cp. genome assembly, complete cp. genome sequences have been comprehensively accepted as valuable and informative data sources for comprehensive evolutionary investigation and have become a highly useful tool to assess the phylogenetic relationships of puzzling groups of angiosperms [15–17]. More cases also confirmed the advantage of the cp. genome in phylogenetic studies at different taxonomic levels. Huang et al. analyzed the cp. genome of *Salix* L. and reconstructed a phylogenetic tree based on the whole cp. genome and common protein-coding sequences of the genus, and the results showed that the genus was monophyletic with high support and was subdivided into two subclades [18]. In Urticeae, cp. genome data proved the monophyly of most genera and provided new insights into the phylogenetic relationship and chloroplast structure evolution [19]. Zhou et al. reconstructed the phylogenetic relationship of Bambusoideae based on cp. genome data and further estimated the divergence time and ancestral distribution, which showed that *Cephalostachyum* Munro and *Schizostachyum* Nees were homologous, and they originated from the early Miocene Eastern Himalayas to northern Myanmar [20].

The taxonomic history of the *Sinodielsia* clade is complex, and its members were enrolled from 17 genera. In phylogenetic studies of these 17 genera, some species were far from other members of genera in phylogeny and clustered in the *Sinodielsia* clade, so these species were adjusted into this clade in the classification system. In this context, we supposed that some members of the *Sinodielsia* clade were inappropriately transferred to this clade because the sampling coverage was not sufficient to detect their proper positions in relevant research. In this study, we sampled 105 cp. genome sequences of 95 species from the *Sinodielsia* clade and its relevant genera and reconstructed their phylogeny based on cp. genome sequences to define the circumscription of the *Sinodielsia* clade and infer the evolutionary history of the clade. This study will promote the comprehension of cp. genome evolution, taxonomy and phylogenetics of Apioideae.

## Results

### Yong Wang ***de novo*** assembled cp. genomes

**Fig. 1:**
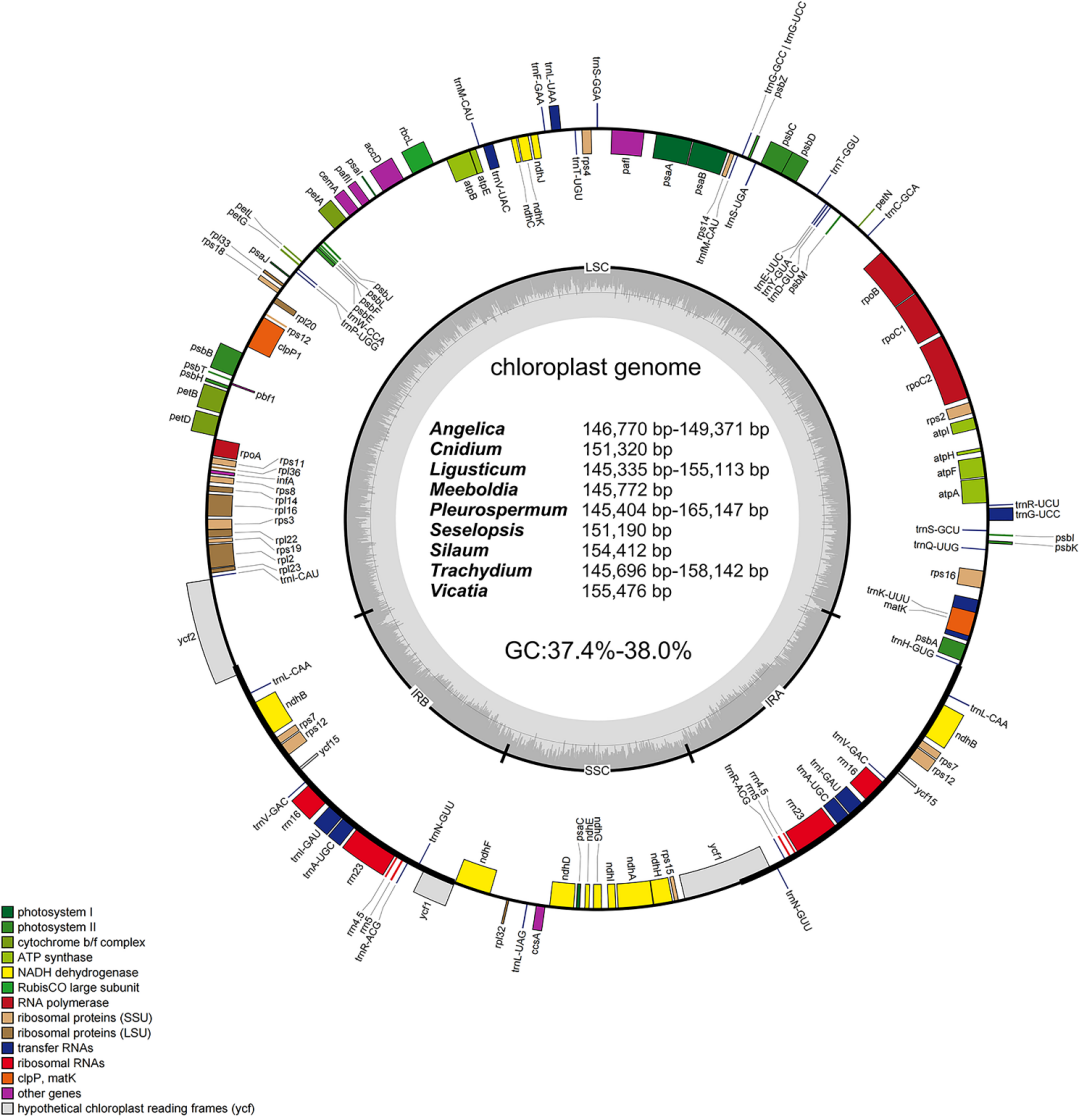
Gene map of cp. genomes of nine *Sinodielsia* Clade relevant genera (the length of each genus was displayed inside). Transcribed clockwise genes are shown outside, while counterclockwise genes are inside. Different functional groups of genes were identified by different colors. The darker gray represents the GC content and the value was displayed inside

Clean reads ranging from 0.81 GB (*Lig. oliverianum*) to 2.75 GB (*A. longicaudata*) of 39 species were extracted from raw reads obtained by the Illumina HiSeq 4,000 system (Table 2). Complete cp. genomes were *de novo* assembled successfully from clean reads and further manually verified to prevent potential assembly errors. Complete cp. genomes of 39 species ranged from 145,335 bp (*Lig. yushuense*) to 165,147 bp (*Ple. foetens*) in length (Table 2). All of them had a highly conserved typical quadripartite structure with two inverted repeat (IR) regions (17,599–31,486 bp), a large single copy (LSC) region (82,048–94,046 bp) and a small single copy (SSC) region (16,343–17,917 bp) (Fig. 1; Table 2). The total GC contents were between 37.4 and 38.0% (Table 2). The total numbers of genes of these 39 complete cp. genomes ranged from 126 (*Ple. hookeri*) to 138 (*Ple. foetens*, *T. tianschanicum*). In detail, these genes included 83–93 protein–coding DNA sequence (CDS) genes, 35–37 transfer RNA (tRNA) and eight ribosomal RNA (rRNA) genes (Table 2). The organization and CDS order (Table S1) of these cp. genomes were highly identical and similar to those of other plants in Apioideae [21–23].

**Fig. 2:**
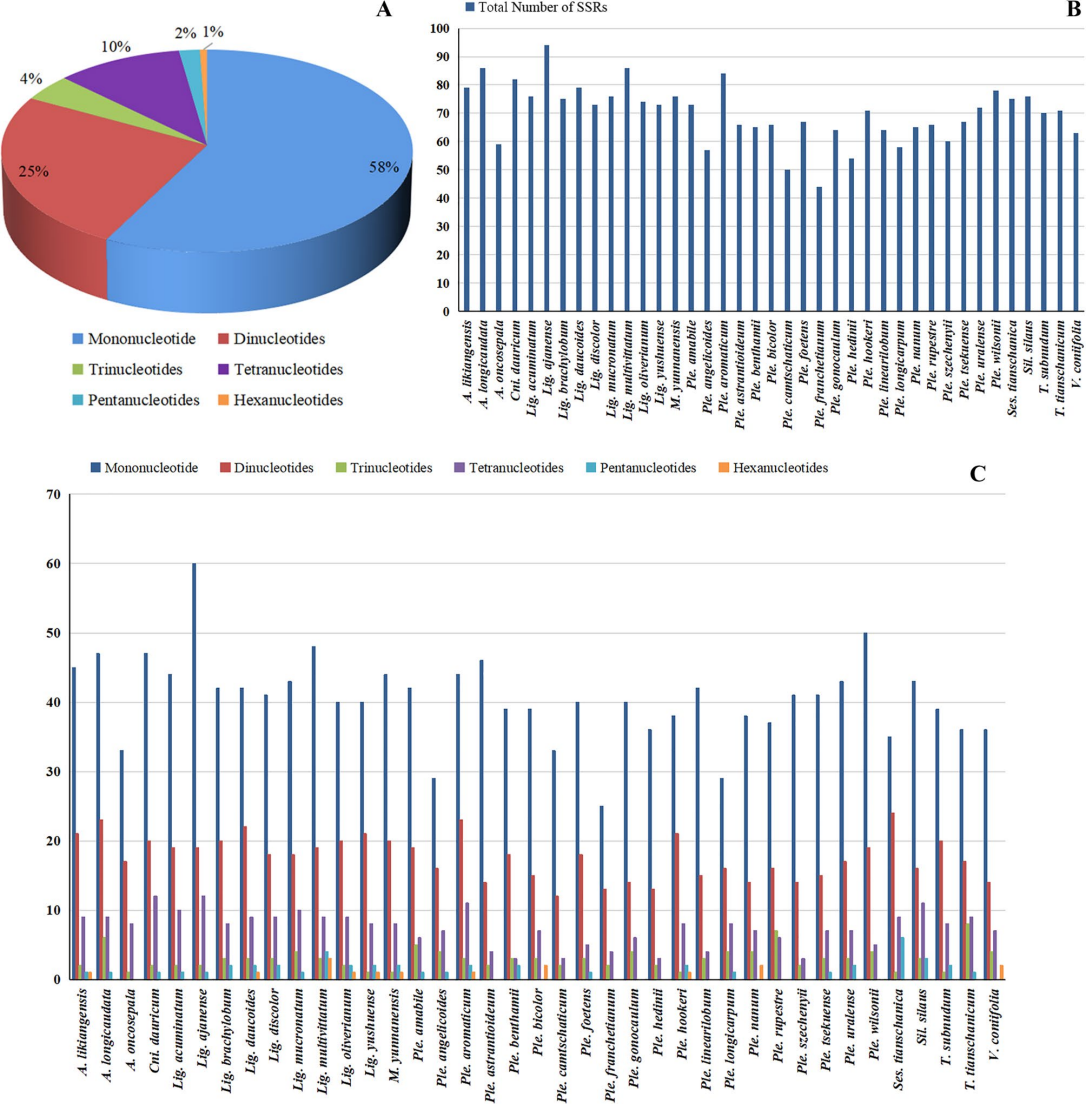
Analysis of simple sequence repeats of 39 species cp. genomes. (**A**) Proportion of different SSRs types; (**B**) Total number of SSRs of 39 species; (**C**) Number of SSRs with different types in 39 species

We detected 2,734 simple sequence repeats (SSRs) among the 39 cp. genomes. Most were mononucleotide repeats (58%), followed by dinucleotides (25%), trinucleotides (4%), tetranucleotides (10%), pentanucleotides (2%) and hexanucleotides (1%) (Fig. 2A). For each genome, the total numbers of SSRs ranged from 44 (*Ple. franchetianum*) to 94 (*Lig. ajanense*) (Fig. 2B). More than half of the SSRs (50.0–69.7%) were mononucleotide repeats in species with newly assembled cp. genomes, except *Ses. tianschanica* (46.7%). (Fig. 2C, Table S2).

**Table 1:** Members of the *Sinodielsia* Clade recorded in references

Genus	Species
*Angelica* Linnaeus	*Angelica sinensis* (Oliv.) Diels ^7^, *A. tianmuensis* Z.H. Pan etT.D. Zhuang ^7^, *A. paeoniifolia* C.Q. Yuan & R.H. Shan ^5^, *A. multicaulis* Pimenov ^10^, *A. ternata* Regel et Schmalh. ^10^, *A. omeiensis* Yuan et Shan ^9^
*Cenolophium* W.D.J. Koch	*Cenolophium denudatum* (Hornem.) Tutin ^5, 8^,
*Conioselinum* Fisch. ex Hoffm.	*Conioselinum tataricum* Hoffm. ^7^, *Con. chinense* (L.) Britton, Sterns & Poggenb. ^5, 8^, *Con. pacificum* (S.Watson) J.M.Coult. & Rose ^8^, *Con. vaginatum* (Spreng.) Thell. ^8^, *Con. cnidiifolium* (Turcz.) A.E.Porsild ^8^
*Cnidium* Cusson	*Cnidium officinale* Makino ^7^, *Cni. dahuricum* (Jacq.) Turcz. ex Fisch. & C.A. Mey. ^5^,
*Levisticum* Hill	*Levisticum officinale* W.D.J. Koch ^7^
*Ligusticum* Linnaeus	*Ligusticum jeholense* (Nakai & Kitag.) Nakai & Kitag. ^7^, *Lig. acuminatum* Franch. ^7^, *Lig. sinense* Oliv. ^7^, *Lig. tenuissimum* (Nakai) Kitag. ^7^, *Lig. chuanxiong* Hort. ^5^, *Lig. pteridophyllum* Franch. ^8^, *Lig. nematophyllum* (Pimenov & Kljuykov) F. T. Pu & M. F. Watson ^8^
*Lithosciadium* Turczaninow	*Lithosciadium multicaule* Turcz. ^7^
*Hymenidium* Lindl.	*Hymenidium apiolens* (C. B. Clarke) Pimenov et Kljuykov ^8^, *H. corydalifolium* (Aitch. & Hemsl.) Pimenov & Kljuykov ^8^, *H. brunonis* Lindl. ^8^
*Paulita* Sojak	*Paulita ovczinnikovii* (Korovin) Sojak ^8^
*Pleurospermum* Hoffm.	*Pleurospermum prattii* H. Wolff ^5^, *Ple. rivulorum* (Diels) K. T. Fu et Y. C. Ho. ^7, 8^
*Prangos* Lindl.	*Prangos haussknechtii* Boiss. ^5^
*Seselopsis* Schischkin	*Seselopsis tianschanica* Schischk. ^7^
*Silaum* Mill.	*Silaum silaus* (Linnaeus) Schinz & Thellung ^8^
*Sinodielsia* H. Wolff	*Sinodielsia delavayi* (Franch.) Pimenov et Kljuykov ^7^
*Sphaenolobium* Pimenov	*Sphaenolobium tianschanicum* (Korovin) Pimenov. ^7^, *Sph. coriaceum* (Korovin) Pimenov ^8^
*Trachydium* Lindl.	*Trachydium subnudum* C. B. Clarke ex H. Wolff ^8^
*Vicatia* DC.	*Vicatia thibetica* H. Boissieu ^5^

^5^ Downie et al. 2010; ^7^ Zhou et al. 2008; ^8^ Zhou et al. 2020; ^9^ Wang et al. 2022; ^10^ Wen et al. 2021

### Phylogenetic relationships of 105 genomes related to the ***Sinodielsia*** clade

**Fig. 3:**
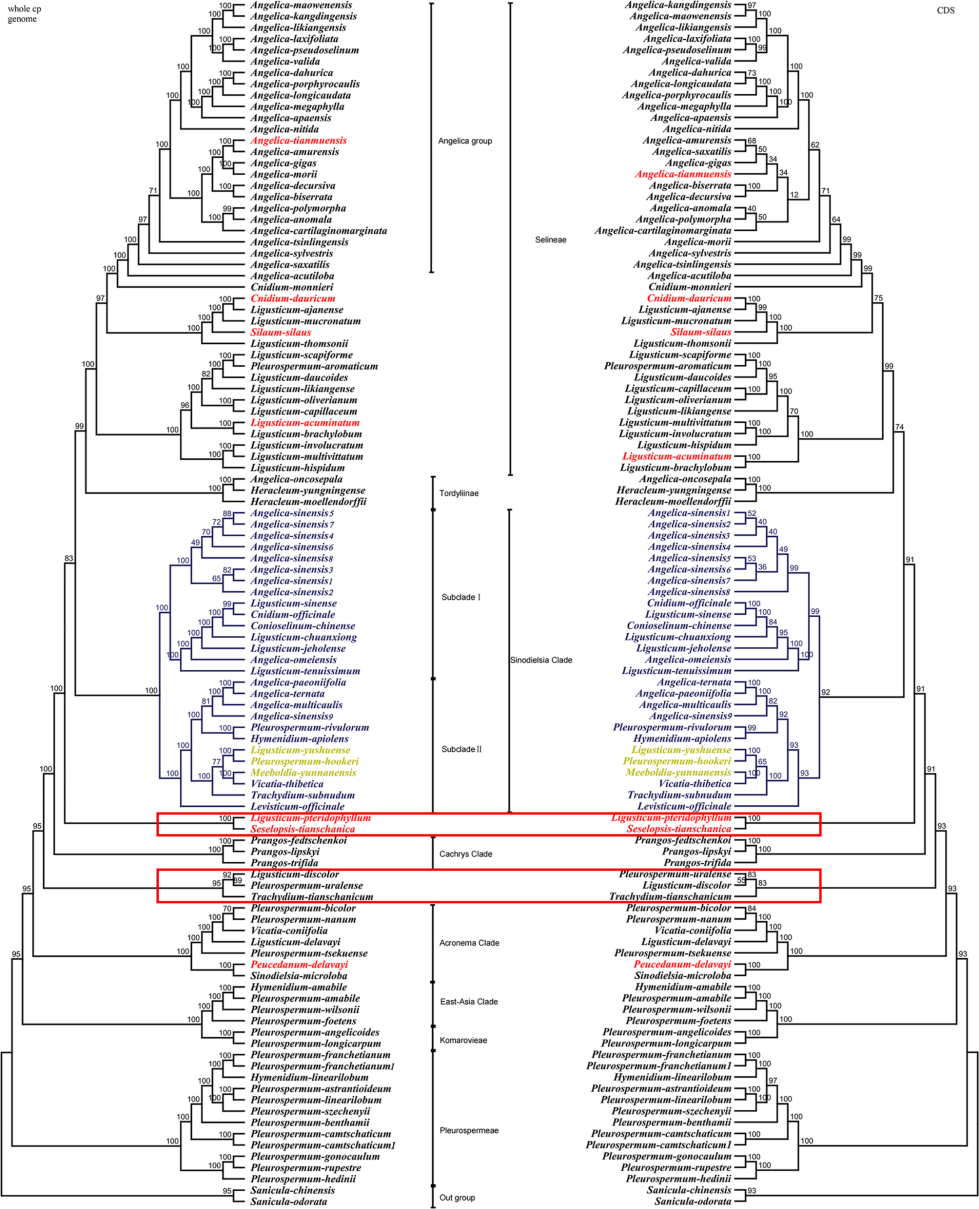
Phylogenetic tree inferred from Maximum-Likelihood based on CDSs and whole cp. genomes of 105 sequences. The numbers were listed at each node represent the bootstrap support (BS) values. The red font labels indicate that the species belonged to *Sinodielsia* Clade in previous studies, and the yellow represents species that were first clustered into *Sinodielsia* Clade

Phylogenetic analyses produced two trees identical in topology based on whole cp. genome and CDS datasets. Our results showed that 100 genomes were clustered into eight clades (Pleurospermeae, *East*–*Asia* clade, Komarovieae, *Acronema* clade, *Cachrys* clade, *Sinodielsia* clade, Tordyliinae, and Selineae), while the other five contained two novel clades, the clade of *Lig. pteridophyllum* and *Ses. tianschanica* and the clade of *Ple. uralense*, *Lig. discolor* and *T. tianschanicum*, respectively (Fig. 3).

**Table 2:** Characteristics of the 39 newly assembled cp. genomes

Species	Clean reads (GB)	Total Length (bp)	LSC Length (bp)	SSC Length (bp)	IR Length (bp)	GC Content (%)	Total genes	CDS	tRNA	rRNA	GenBank Accession No.
*A. likiangensis*	1.81	146,770	94,046	17,526	17,599	37.5	129	85	36	8	OP672440
*A. longicaudata*	2.75	146,976	93,631	17,707	17,819	37.5	129	85	36	8	OP672441
*A. oncosepala*	1.45	149,371	92,212	17,517	19,821	37.5	129	84	37	8	OP672442
*Cni. dauricum*	1.26	151,320	89,529	17,425	22,183	37.6	129	85	36	8	OP672443
*Lig. acuminatum*	2.09	148,510	92,245	17,587	19,339	37.4	129	85	36	8	OP672444
*Lig. ajanense*	1.90	150,983	89,216	17,449	22,159	37.6	130	87	36	8	OP672445
*Lig. brachylobum*	1.74	148,500	92,340	17,608	19,276	37.4	129	85	36	8	OP672446
*Lig. daucoides*	1.51	148,200	92,251	17,679	19,135	37.5	129	85	36	8	OP672447
*Lig. discolor*	1.01	155,113	85,125	17,504	26,242	37.8	133	88	37	8	OP672448
*Lig. mucronatum*	1.01	147,768	93,099	17,561	18,554	37.6	129	85	36	8	OP672449
*Lig. multivittatum*	1.48	148,262	91,571	17,631	19,530	37.4	129	85	36	8	OP672450
*Lig. oliverianum*	0.81	147,799	91,902	17,503	19,197	37.5	129	85	36	8	OP672451
*Lig. yushuense*	1.00	145,335	92,241	17,454	17,830	37.7	128	85	35	8	OP672452
*M. yunnanensis*	1.60	145,772	92,155	17,463	18,077	37.7	128	85	35	8	OP672453
*Ple. amabile*	2.21	155,882	85,601	17,573	26,354	37.7	133	88	37	8	OP672454
*Ple. angelicoides*	1.21	156,304	85,916	17,894	26,247	37.7	133	88	37	8	OP672455
*Ple. aromaticum*	1.52	148,074	92,175	17,547	19,176	37.5	129	85	36	8	OP672456
*Ple. astrantioideum*	1.38	155,972	85,688	17,798	26,243	37.9	133	88	37	8	OP672457
*Ple. benthamii*	1.87	155,425	85,223	17,778	26,212	38.0	131	86	37	8	OP672458
*Ple. bicolor*	1.62	155,294	84,762	16,688	26,922	37.6	133	88	37	8	OP672459
*Ple. camtschaticum*	1.08	154,379	84,908	17,493	25,989	38.0	131	86	37	8	OP672460
*Ple. foetens*	1.42	165,147	85,223	16,952	31,486	37.7	138	93	37	8	OP672461
*Ple. franchetianum*	1.59	155,674	85,761	17,743	26,085	38.0	131	86	37	8	OP672462
*Ple. gonocaulum*	1.07	155,399	85,199	17,760	26,220	37.9	133	88	37	8	OP672463
*Ple. hedinii*	1.98	156,155	85,648	17,767	26,370	37.9	133	88	37	8	OP672464
*Ple. hookeri*	0.97	145,404	92,267	17,475	17,831	37.6	126	83	35	8	OP672465
*Ple. linearilobum*	1.27	155,979	85,707	17,788	26,242	37.9	131	86	37	8	OP672466
*Ple. longicarpum*	1.08	156,464	86,037	17,917	26,255	37.7	131	86	37	8	OP672467
*Ple. nanum*	2.07	155,709	84,912	16,343	27,227	37.6	133	88	37	8	OP672468
*Ple. rupestre*	1.00	155,917	85,617	17,802	26,249	37.9	133	88	37	8	OP672469
*Ple. szechenyii*	1.91	156,023	85,714	17,795	26,257	37.9	131	86	37	8	OP672470
*Ple. tsekuense*	1.79	155,231	84,777	16,704	26,875	37.6	133	88	37	8	OP672471
*Ple. uralense*	1.37	155,483	83,707	17,488	27,144	37.8	135	90	37	8	OP672472
*Ple. wilsonii*	1.20	155,950	85,581	17,705	26,332	37.7	131	86	37	8	OP672473
*Ses. tianschanica*	1.74	151,190	90,683	17,537	21,485	37.6	130	86	36	8	OP672474
*Sil. silaus*	1.64	154,412	86,816	17,488	25,054	37.4	130	85	37	8	OP672475
*T. subnudum*	1.07	145,696	92,111	17,453	18,066	37.7	129	85	36	8	OP672476
*T. tianschanicum*	1.11	158,142	82,048	17,070	29,512	37.7	138	93	37	8	OP672477
*V. coniifolia*	2.21	155,476	84,927	16,689	26,930	37.6	133	88	37	8	OP672478

Nineteen species from 10 genera were clustered into the *Sinodielsia* clade. It was close to Selinese and Tordyliinae (BS = 83/91) in topology, which was in line with previous studies of Apioideae based on both ITS and cp. genome data [5, 6, 10]. The clade was divided into two subclades with strong bootstrap values. One subclade included *A. sinensis* (8 individuals), *Cni. officinale*, *Lig. sinense*, *Con. chinense*, *Lig. chuanxiong*, *Lig. jeholense*, *A. omeiensis* and *Lig. tenuissimum* (Fig. 3, subclade I, BS = 99/100), and another subclade comprised 12 other species, namely, *A. ternata*, *A. paeoniifolia*, *A. multicaulis*, *(A) sinensis* (1 individual), *Ple. rivulorum*, *H. apiolens*, *L. yushuense* J. T. Pan, *Ple. hookeri* C. (B) Clarke, *Meeboldia yunnanensis* (H. Wolff) Constance & F. T. Pu, *V. thibetica*, *T. subnudum* and *Lev. officinale* (Fig. 3, subclade II, BS = 93/100).

### Comparing the IR boundaries of the ***Sinodielsia*** clade

**Fig. 4:**
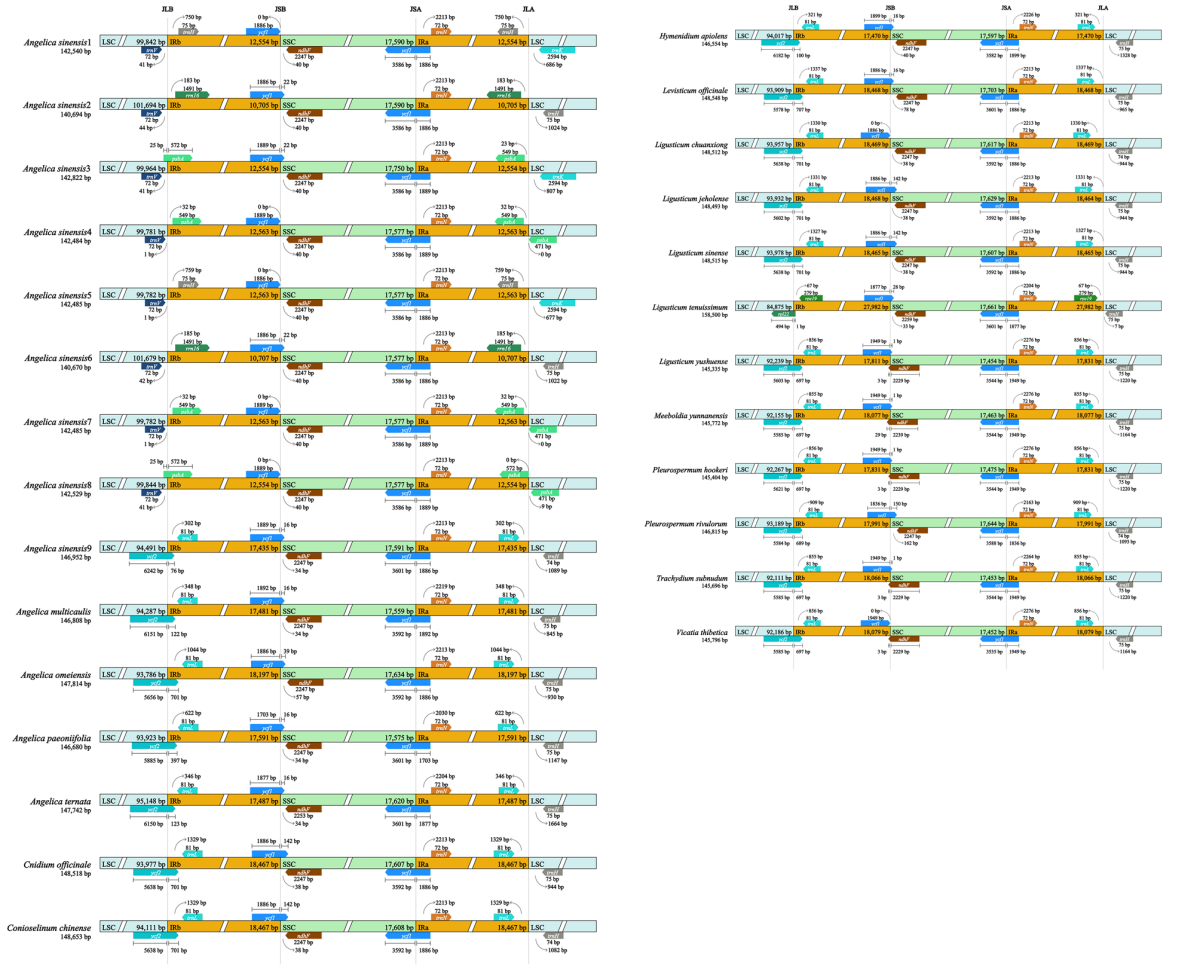
Comparison of LSC, SSC, IRs region boundaries of 27 genomes within the *Sinodielsia* Clade. The figure is not drawn to scale

Chloroplast genomes were highly conserved in structure and size, while the change in the location of the IR/SC junction was due to the universally existing expansion and shrinkage of the IR regions [24, 25]. Comparison of IR boundaries among 27 genomes of the *Sinodielsia* clade displayed diverse expansion and contraction of the IR regions (Fig. 4). The junction site of LSC/IRb (JLB) was located in the *ycf*2 gene in 18 out of 27 genomes and extended to the *rpl*22 gene in the *Lig. tenuissimum* genome. For eight *A. sinensis* genomes (*A. sinensis*1–*A. sinensis*8), it departed 1–44 bp from the *trn*V gene. Among these 27 genomes, all the junction sites of IRb/SSC (JSB) were close (within 33–162 bp) to or located in the *ndh*F gene. The junction sites of SSC/IRa (JSA) were located in the *ycf*1 gene in the 27 genomes. The junction sites of IRa/LSC (JLA) were near the *trn*H gene (7–1664 bp) in 23 genomes except the cp. genomes from four individuals of *A. sinensis* (*A. sinensis*3, *A. sinensis*4, *A. sinensis*7, *A. sinensis*8), whose boundaries extended 0–32 bp into the *psb*A gene.

### Comparative genomic analysis in the ***Sinodielsia*** clade

**Fig. 5:**
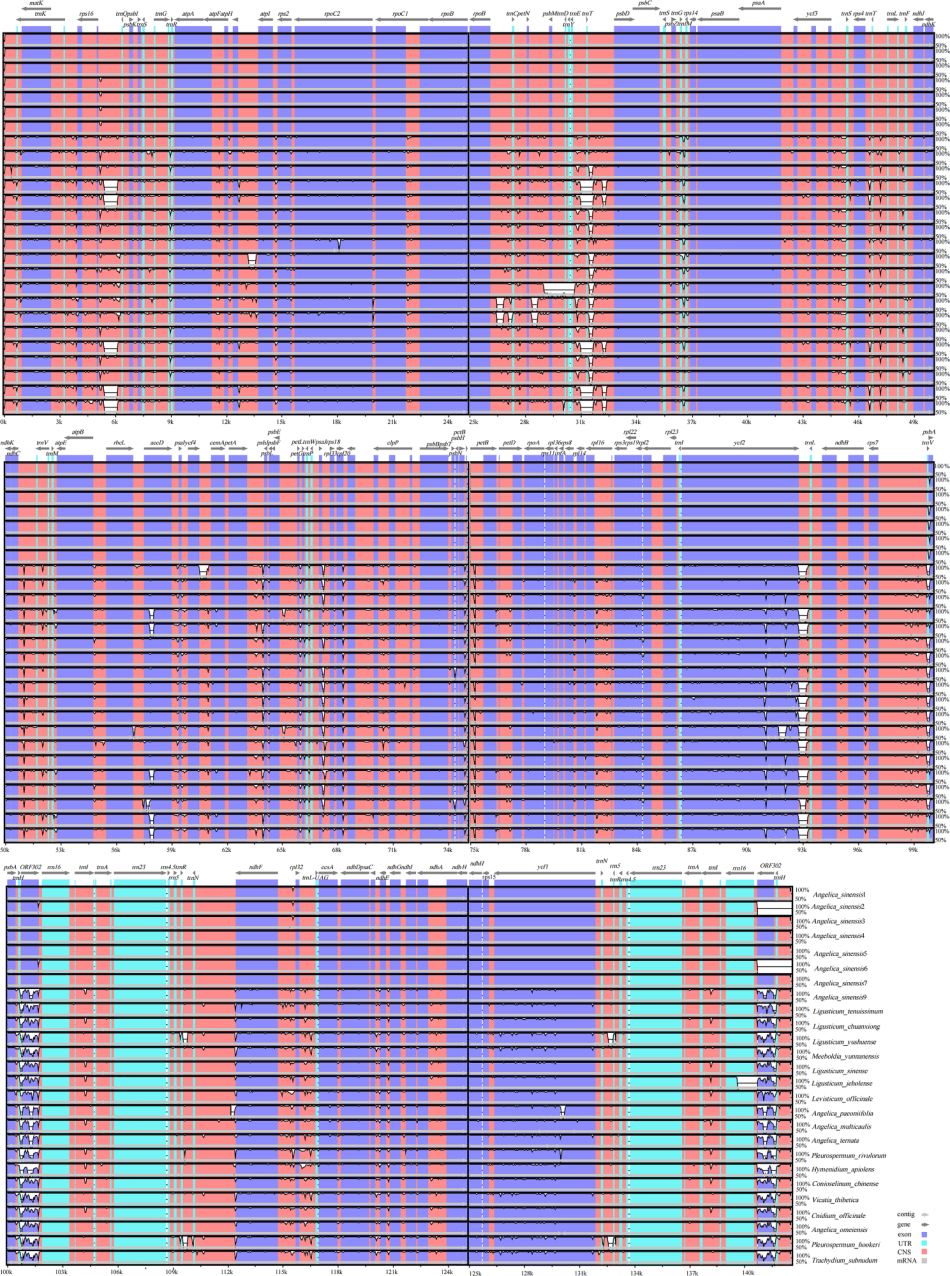
Twenty-seven sequences alignment was performed by mVISTA using *Angelica sinensis*8 as a reference. The vertical scale represents the percentage of identity, ranging from 50 to 100%

**Fig. 6:**
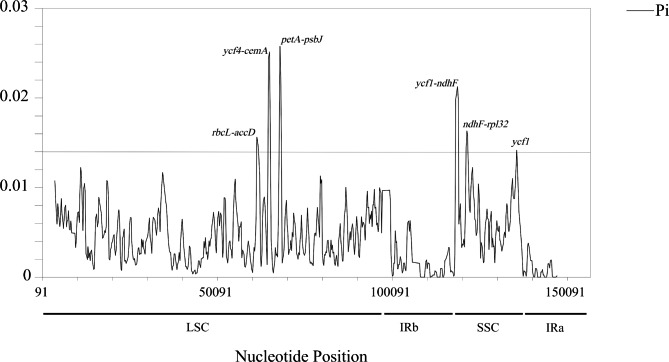
The DnaSP graph of nucleotide diversity (Pi) value of the 27 cp. genomes in *Sinodielsia* Clade

Using the mVISTA program and referring to *A. sinensis*8 (MK688991), the sequence identity analysis revealed more sequence mutations in noncoding regions than in coding regions in the *Sinodielsia* clade (Fig. 5). Furthermore, sliding window analysis showed that the nucleotide diversity (Pi) values of these 27 genomes ranged from 0 to 0.02576 (Fig. 6). The average Pi value was 0.00476 in the LSC regions and 0.00706 in the SSC regions. In contrast, the average Pi values of the IR regions were the lowest (0.00154). We observed six mutation hotspots (highly variable regions) with Pi values over 0.01400, including five noncoding regions and one gene region (Fig. 6). Of them, three noncoding regions were located in the LSC, and they were *rbc*L–*acc*D (Pi, 0.01456–0.01563), *ycf*4–*cem*A (Pi, 0.01570–0.02514) and *pet*A–*psb*J (Pi, 0.01538–0.02576). The SSC region contributed the other three mutation hotspots, including two noncoding regions and a gene, which were *ycf*1–*ndh*F (Pi, 0.01985–0.02127), *ndh*F–*rpl*32 (Pi, 0.01444–0.01632) and *ycf*1 (Pi, 0.01418), respectively. Furthermore, *ndh*F–*rpl*32 and *ycf*1 were highly variable in all 105 cp. genomes (Fig. S1).

**Fig. 7:**
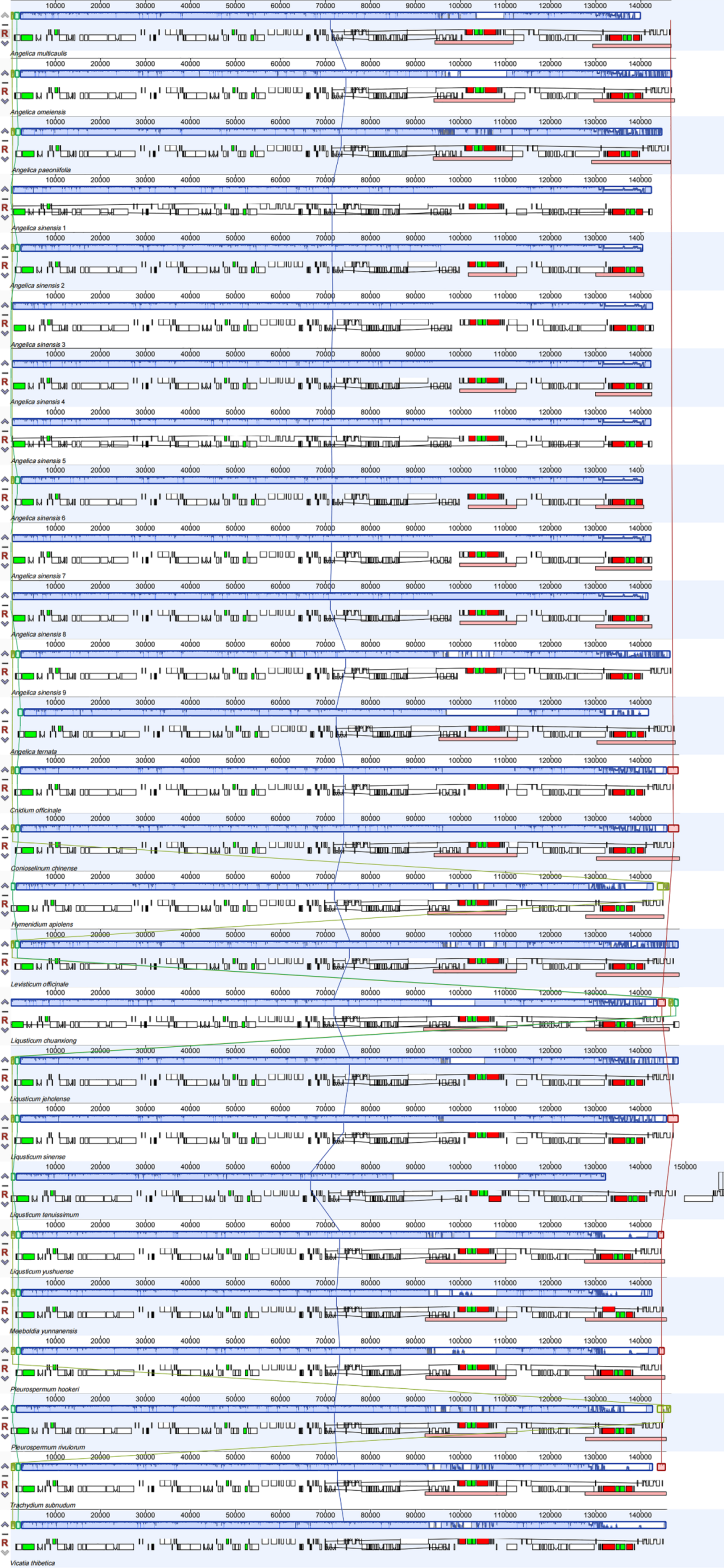
Mauve alignment of the complete cp. genome of 27 *Sinodielsia* Clade species. The strip structure with the same color in the figure is a local collinear block, representing a set of homologous genes. The strip area below the horizontal line of each genome indicates that inversion has occurred

Comparative analysis of cp. genome sequences using the Mauve alignment approach showed that the genome structures of the *Sinodielsia* clade species were conservative, and no potential rearrangement or change was detected in gene order (Fig. 7).

## Discussion

### Genome features

A total of 105 complete cp. genomes from 16 genera were different in size, ranging from 140,670 bp (*A. sinensis*) to 165,147 (*Ple. foetens*) (Table S3), which showed that the cp. genomes in these species had distinct characteristics. Nevertheless, species within the *Sinodielsia* clade had a moderate-length genome, and their lengths ranged from 145,335 bp to 148,653 bp, except for the minimum length of *A. sinensis* (8 individuals, 140,670 bp–142,822 bp) and the maximum length of *Lig. tenuissimum* (158,500 bp). Notably, *Pleurospermum* had a large average cp. genome size of 154,687 bp, but two species of this genus, *Ple*. *rivulorum* and *Ple*. *hookeri*, were clustered in the *Sinodielsia* clade, and the cp. genomes of both species were relatively short in size (146,815 bp and 145,400 bp, respectively).

All 105 cp. genomes had a typical quadripartite structure and were conserved in gene order, similar to other angiosperm cp. genomes [14]. However, many genes of the cp. genome have been lost in different plants [26–30], such as *acc*D, *ycf*1, *inf*A, *clp*P, *ccs*A, *rps*12, *rps*16, and *rpl*23. In this study, the gene number of 105 cp. genomes ranged from 121 to 144, and *rps*12, *rps*16, *ycf*15 and *ycf*1 were frequently missing from these cp. genomes (Table S1 and Table S3). The phenomenon of these gene losses in Apioideae was probably a result of extensive hybridization and/or cp. genome decay within various lineages [28, 31].

### Circumscription and phylogeny of the ***Sinodielsia*** clade

In the present study, 105 taxa were sampled from 16 genera related to the *Sinodielsia* clade, and their phylogeny was constructed based on whole cp. genome sequences and CDSs. We sampled not only species of the *Sinodielsia* clade but also extensive relevant taxa, which covered eight out of 30 major clades of Apioideae (e.g., Pleurospermeae, *East*–*Asia* clade, Komarovieae, *Acronema* clade, *Cachrys* clade, *Sinodielsia* clade, Tordyliinae, and Selineae). The relationships of eight clades inferred based on cp. genome data were consistent with those in previous studies [5, 6, 10, 12], except for two novel clades, the clade of *Lig. pteridophyllum* and *Ses. tianschanica*, and the clade of *Ple. uralense*, *Lig. discolor* and *T. tianschanicum*.

Our results confirmed that the *Sinodielsia* clade was the sister to the cluster of Selineae and Tordyliinae, but the circumscription of the *Sinodielsia* clade was different from the results of previous studies [5, 7–10]. Three species of the *Sinodielsia* clade, *Lig. yushuense*, *Ple. hookeri* and *M. yunnanensis*, were first clustered into this clade, and it seemed that they were new members of the *Sinodielsia* clade. Nevertheless, we found that seven species formerly accommodated in the *Sinodielsia* clade were excluded from this clade. *Lig. pteridophyllum* and *Ses. tianschanica* formed an independent clade departing from the *Sinodielsia* clade, and it was the sister to the large cluster of three clades, Selineae, Tordyliinae and *Sinodielsia*. *A. tianmuensis* was clustered in the *Angelica* group. *Peu. delavayi* was nested in the *Acronema* clade, and the other three species were in Selineae, *Cni. dauricum*, *Lig. acuminatum* and *Sil. silaus*.

The *Sinodielsia* clade was established, and its members were enrolled based on ITS, but the positions of the aforementioned seven species were not supported by cp. data. Conflicts between results from cp. DNA and nrDNA data probably reflected complementary processes of speciation in diverse inheritance patterns [32]. Additionally, some genetic events might contribute to these conflicts in phylogeny, including incomplete lineage sorting, hybridization/introgression, paralogy, gene duplication and/or loss, and horizontal gene transfer [33, 34]. Positions of *Peu. delavayi*, *Lig. pteridophyllum* and *Ses. tianschanica* were unstable in different studies when they were investigated together with different species and genera based on ITS [5, 7, 8, 13], while they were not clustered in the *Sinodielsia* clade on the phylogenetic trees based on cp. genome data. We speculated that the lack of relatives resulted in clustering of these species in the *Sinodielsia* clade in studies based on ITS, and conflicts between results from cp. DNA and nrDNA could be attributed to low sampling coverage in previous studies. We have no direct evidence to clarify this phenomenon in *A. tianmuensis*, *Cni. dauricum*, *Lig. acuminatum* and *Sil. silaus*, but we preferred to attribute the conflicts to hybridization or introgression after comparing them with their relatives in terms of morphology and distribution.

Except for cultivated and introduced species, all species in the *Sinodielsia* clade gathered into two subclades relevant to geographical distributions. Species of subclade I were widely distributed in Asia and Europe, except *A. omeiensis* in Emei Mountain, Sichuan Province. *A. omeiensis* is a poorly known specieswith reputed medicinal properties, which is recorded only from a few collections. Recent research suggests that it is conspecific with *A. wilsonii* and *A. sinensis* var. *wilsonii* [35]. Species of subclade II were distributed in Western China except for the introduced *Lev. officinale* and one individual of cultivated *A. sinensis*. *A. ternata* and *A. multicaulis* were distributed in Xinjiang Province, and *Lig. yushuense* was in Qinghai Province. The other seven species were endemic to the Himalaya region, *A. paeoniifolia*, *Ple. rivulorum*, *H. apiolens*, *Ple. hookeri*, *M. yunnanensis*, *V. thibetica* and *T. subnudum*.

*Angelica sinensis* is a famous and widely used Chinese traditional medicine herb, and it has been cultivated for more than 1,000 years. We sampled nine individuals of *A*. *sinensis* from different plant areas, but eight of them were clustered in subclade I and one in subclade II. This result seems provide a hint of its multiple domestication, and this study pointed out further direction on the investigation of the cultivation origin of *A. sinensis*.

## Potential markers for molecular identification and phylogeny of apiaceae at low taxonomic levels

Higher Pi values indicated more mutations and higher evolutionary rates in highly variable regions than in other regions [36]. Multiple variable regions have been identified in angiosperms [37–39]. Unfortunately, these regions often lack variations in closely related species in Apiaceae, especially those diverged recently in evolutionary history, so that there are very few choices in chloroplast DNA markers suitable for phylogeny of Apiaceae at low taxonomic levels, and research on molecular systematics of the family relies heavily on the use of ITS [5, 8, 11–13]. In the present study, we scanned the whole cp. genomes of the *Sinodielsia* clade and detected six mutation hotspot regions in both noncoding regions and CDS, *rbc*L–*acc*D, *ycf*4–*cem*A, *pet*A–*psb*J, *ycf*1–*ndh*F, *ndh*F–*rpl*32 and *ycf*1. Among the six highly variable regions, high variability of *ndh*F*–rpl*32 and *ycf*1 was also observed in sliding window analysis of 105 whole cp. genomes. These six regions, especially *ndh*F*–rpl*32 and *ycf*1, were highly variable and should be the first consideration as screening suitable loci to distinguish closely related species or genera in identification and phylogenetic analyses of the *Sinodielsia* clade, even Apioideae.

## Conclusion

The *Sinodielsia* clade has been an incomprehensible group of Apiaceae in terms of taxonomy, and its members are tangled with 16 genera from different clades in morphology. In this study, we assembled complete cp. genomes of 39 species relative to the *Sinodielsia* clade and scanned genome characteristics in terms of genome size, GC content, SC/IR boundaries, gene number, repeat types and distribution. Then, we performed phylogenetic analysis based on 105 cp. genome sequences from 16 genera relative to the *Sinodielsia* clade. The phylogenetic analysis showed that 19 species were clustered into the *Sinodielsia* clade, and the clade was subdivided into two subclades relevant to geographical distributions, except cultivated and introduced species. Six mutation hotspot regions were detected from the whole cp. genomes among the *Sinodielsia* clade; namely, *rbc*L–*acc*D, *ycf*4–*cem*A, *pet*A–*psb*J, *ycf*1–*ndh*F, *ndh*F–*rpl*32 and *ycf*1, and *ndh*F–*rpl*32 and *ycf*1 were highly variable in the 105 sampled cp. genomes. These mutation hotspot regions could be used as potential DNA markers in identification and phylogenetic analyses of the *Sinodielsia* clade and Apioideae. Our study provided new insights into the phylogeny of the *Sinodielsia* clade and provided valuable information on cp. genome evolution in Apioideae.

## Methods

### Taxon sampling

We sequenced and assembled the cp. genomes of 39 species from nine genera relevant to the *Sinodielsia* clade. Samples were collected from the National Wild Plant Germplasm Resource Center and Herbarium of Kunming Institute of Botany, and we used them following the prescribed procedures of the Kunming Institute of Botany. Vouchers were preserved in the Herbarium of Kunming Institute of Botany (KUN), and Shaotian Chen reviewed and identified the vouchers. To infer the phylogeny of the *Sinodielsia* clade, we downloaded published cp. genome sequences of 66 species related to the *Sinodielsia* clade from GenBank (https://www.ncbi.nlm.nih.gov/genbank/). In total, 105 sequences from 23 species from 12 genera were members of the *Sinodielsia* clade. Details of 39 newly sequenced samples and 66 published sequences are shown in the supporting information (Table S4 and Table S5).

### DNA extraction and cp. genome sequencing

Total genomic DNA was extracted following a modified CTAB protocol [40]. Genomic DNA from each sample was subsequently assessed for quality using both a NanoDrop 2000 spectrophotometer (Thermo Fisher Scientific, United States) and agarose gel electrophoresis before library preparation. The libraries were generated using the NEBNext Ultra II DNA Library Prep Kit for Illumina following the manufacturer’s instructions. Sequencing was performed on the Illumina HiSeq 4000 platform with 150 bp paired–end reads. The obtained raw reads were adapter–trimmed and quality–filtered by AdapterRemoval v2 (trimwindows = 5 and minlength = 50) [41]. Clean reads were deposited in GSA (Genome Sequence Archive, https://ngdc.cncb.ac.cn/gsa/, Accession No.: CRA007981, CRA006303).

### Chloroplast genome assembly and annotation

Clean reads were qualitatively evaluated and assembled using GetOrganelle version 1.7.4 [42]. Assembled circular complete cp. genomes were checked and aligned with the reference to complete cp. genomes of *Lig*. *sinense* (MN652884) and *A*. *sinensis* (MW820164) using Geneious version 2022.0.1 [43]. The online program GeSeq (https://chlorobox.mpimp–golm.mpg.de/geseq.html) was used to annotate the complete cp. genomes [44]. The annotated genomes were further examined using Geneious version 2022.0.1 to prevent potential annotation errors. The online program OGDRAW (https://chlorobox.mpimp–golm.mpg.de/OGDraw.html) was used to plot the gene maps of complete cp. genomes [45]. Annotated cp. genomes were deposited in GenBank (Accession No.: OP672440–OP672478, Table 2).

### Simple sequence repeat analysis

Simple sequence repeats (SSRs) were searched using the online program MISA [46] (https://webblast.ipk–gatersleben.de/misa/). The program parameters were set as follows: minimum numbers of repetitions for mononucleotide SSRs, dinucleotide repeat SSRs, trinucleotide repeat SSRs, tetra–, penta–, and hexanucleotide repeat SSRs were 10, 5, 4, 3, 3 and 3, respectively.

### Sequence characteristics analysis

Geneious version 2022.0.1 was used to count genome sizes, GC contents, LSC/SSC/IR lengths and gene numbers [43]. The IR/SC boundaries of the *Sinodielsia* clade species were compared to describe IR expansion and contraction. Whole cp. genome alignment was performed and visualized by the online tool mVISTA [47] (http://www.gsd.lbl.gov/vista/mvista/). Nucleotide divergence values were computed by DnaSP version 6.12.03 [48]. The parameters of the sliding window method were set to a step size of 200 bp and a window length of 600 bp. Comparative analysis of cp. genome structure and gene rearrangements was performed by Geneious version 2022.0.1 using plague Mauve alignment [49].

### Sequence alignment and phylogenetic analysis

Phylogenetic trees were constructed based on two datasets of CDS and whole cp. genome sequences of 105 complete cp. genomes, and two species (*Sanicula chinensis* Bunge, *Sa. odorata* (Raf.) K.M. Pryer et L.R. Phillippe) were assigned as outgroups to root trees. To avoid calculating the same information twice, the CDSs in the second inverted repeat region were eliminated from the CDS dataset. Both datasets were aligned by MAFFT version 7.490 [50] and adjusted automatically using TBtools version 1.09876 [51]. Maximum likelihood (ML) trees were constructed for each of the two datasets using IQ–tree version 2.2.0 under a GTR + I + G + F4 model with 1000 bootstrap replicates [52]. The ML tree file was imported into MEGA11 to view and edit the tree, and the vector graph of output trees was saved to the file [53].

## Electronic supplementary material

Below is the link to the electronic supplementary material.

Supplementary Material 1

Supplementary Material 2

Supplementary Material 3

Supplementary Material 4

Supplementary Material 5

Supplementary Material 6

Supplementary Material 7

## Data Availability

New sequenced and other published chloroplast genome sequences can be found in GenBank (https://www.ncbi.nlm.nih.gov/genbank/), and the accession numbers are shown in Table 1.
