# Supplementary material for: Chloroplast genome characteristics and phylogeny of the sinodielsia clade (apiaceae: apioideae)
Source: BMC Plant Biol. 2023 May 29;23:284. doi: 10.1186/s12870-023-04271-2 (PMC10226202; doi:10.1186/s12870-023-04271-2)
Supplement: Supplementary file 1 — Supplementary Material 1 [file 12870_2023_4271_MOESM1_ESM.docx]

**Supplementary Fig. S1.** The DnaSP graph of nucleotide diversity (Pi) value of the 105 cp genomes in *Sinodielsia* Clade and its related species.
