# Supplementary figures and images for: Chloroplast genome characteristics and phylogeny of the sinodielsia clade (apiaceae: apioideae)
Source: BMC Plant Biol. 2023 May 29;23:284. doi: 10.1186/s12870-023-04271-2 (PMC10226202; doi:10.1186/s12870-023-04271-2)

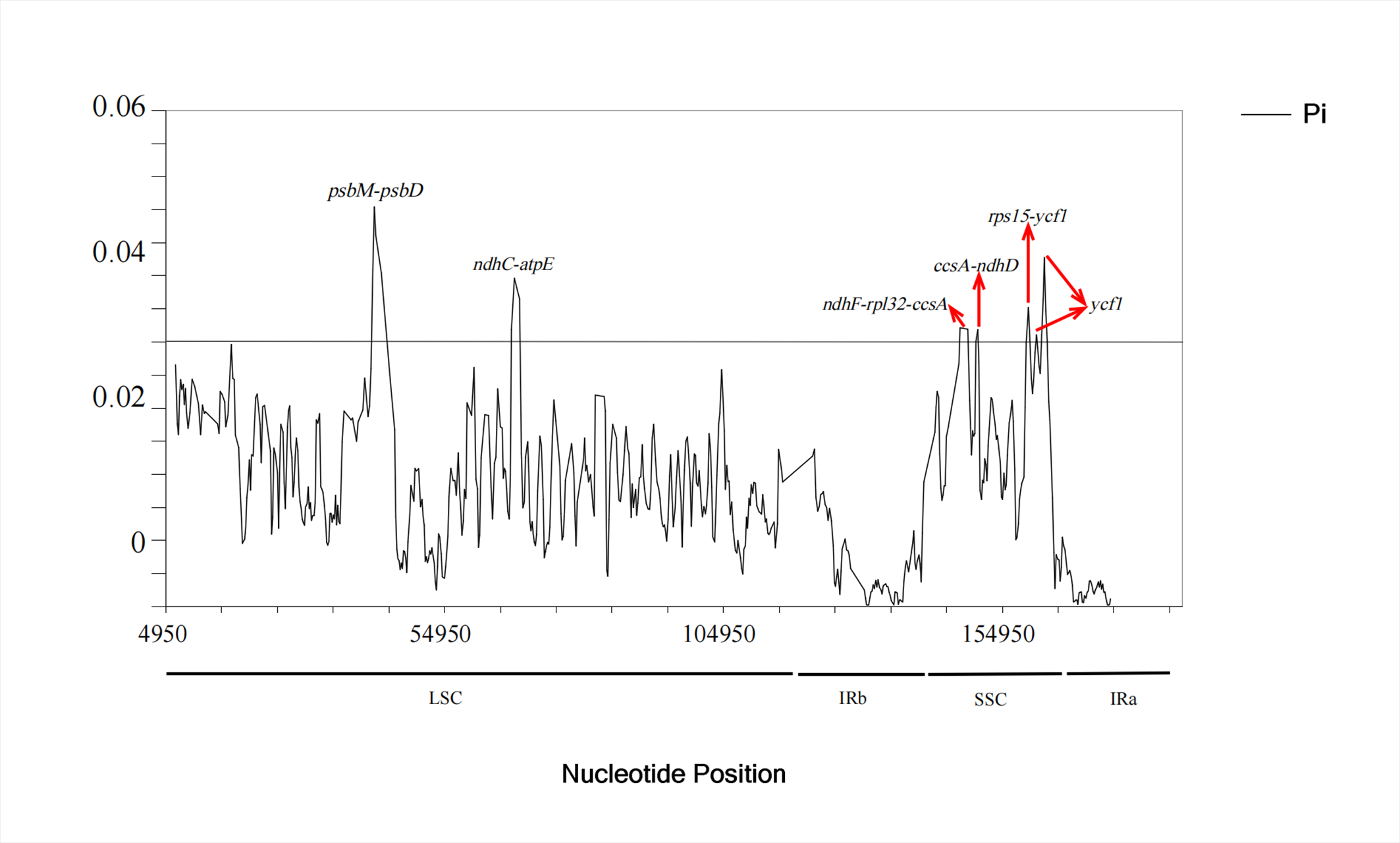

Supplement: Supplementary file 7 — Supplementary Material 7 [file 12870_2023_4271_MOESM7_ESM.tif]
